# Decreased percentage of CD4^+^Foxp3^+^TGF-β^+^ and increased percentage of CD4^+^IL-17^+^ cells in bronchoalveolar lavage of asthmatics

**DOI:** 10.1186/1476-9255-11-22

**Published:** 2014-08-09

**Authors:** Adam Barczyk, Wladyslaw Pierzchala, Gaetano Caramori, Ryszard Wiaderkiewicz, Marcin Kaminski, Peter J Barnes, Ian M Adcock

**Affiliations:** 1 Katedra i Klinika Pneumonologii, Slaski Uniwersytet Medyczny w Katowicach, Katowice, Poland; 2 Centro Interdipartimentale per lo Studio delle Malattie Infiammatorie delle Vie Aeree e Patologie Fumo-correlate (CEMICEF; formerly Centro di Ricerca su Asma e BPCO), Sezione di Malattie dell’Apparato Respiratorio, Università di Ferrara, Ferrara, Italy; 3 Katedra i Zaklad Histologii i Embriologii, Slaski Uniwersytet Medyczny w Katowicach, Katowice, Poland; 4 Airway Disease Section, National Heart and Lung Institute, Imperial College London, Dovehouse Street, London SW3 6LY, UK

**Keywords:** Asthma, BAL, CD4^+^IL-17^+^ cells, CD4^+^Foxp3^+^TGF-β^+^ cells

## Abstract

**Background:**

Asthma is a chronic inflammatory disorder of the airways with the proven role of Th2 cells in its pathogenesis. The role and characteristic of different subsets of CD4^+^ cells is much less known.

**Aim:**

The aim of the study was to analyze the incidence of different subsets of CD4^+^ T cells, in particular different subsets of CD4^+^ cells with the co-expression of different cytokines.

**Methods:**

Twenty five stable asthmatic and twelve age-matched control subjects were recruited to the study. Bronchoscopy and bronchoalveolar lavage (BAL) were performed in all study subjects. CD4^+^ T cells were isolated from BAL fluid by positive magnetic selection. After stimulation simultaneous expression of TGF-β, FoxP3, CD25, IFN-γ, IL-4, TNF-α (set 1); IL-10, FoxP3, CD25, IFN-γ, IL-4, MIP-1β (set 2); IL-17A, IL-8, IFN-γ, IL-4, MIP-1β (set 3) were measured by flow cytometry.

**Results:**

The percentage of CD4^+^ cells co-expressing Foxp3 and TGF-β (CD4^+^Foxp3^+^TGF-β^+^ cells) was significantly lower (*P* = 0.03), whereas the percentage of CD4^+^IL-17^+^ cells (*P* = 0.008), CD4^+^IL-17^+^ IFN-γ^+^ cells (*P* = 0.047) and CD4^+^IL-4^+^ cells (*P* = 0.01) were significantly increased in asthmatics compared with that seen in healthy subjects. A significantly higher percentage of CD4^+^Foxp3^+^ cells from asthma patients expressed IFN-γ (*P* = 0.01), IL-4 (*P* = 0.004) and CD25 (*P* = 0.04), whereas the percentage of CD4^+^IL-10^+^ cells expressing Foxp3 was significantly decreased in asthmatics (*P* = 0.03). FEV_1_% predicted correlated negatively with the percentage of CD4^+^IL-17^+^ cells (r = -0.33; *P* = 0.046) and positively with CD4^+^Foxp3^+^TGF-β^+^ cells (r = 0.43; *P* = 0.01).

**Conclusions:**

Our results suggest that in the airways of chronic asthma patients there is an imbalance between increased numbers of CD4^+^IL-17^+^ cells and Th2 cells and decreased number of CD4^+^Foxp3^+^TGF-β^+^.

## Background

Asthma is a chronic inflammatory disorder of the airways characterized by variable airflow obstruction and presence of airway hyperresponsiveness that leads to typical symptoms like recurrent episodes of wheezing, breathlessness, chest tightness and coughing [1]. CD4^+^ T cells are one of the most important cells in the pathogenesis of airway inflammation in asthma. CD4^+^ T cells are traditionally divided into Th1 and Th2 subsets based on profile of their cytokines production [2,3]. Th2 cells, which are characterized by IL-4, IL-5, IL-9 and IL-13 cytokine production, predominate in asthma, whereas Th1 cells producing IFN-γ, IL-2 and TNF have rarely been associated with asthma [2,3]. More recently new subsets of CD4^+^ T cells have been described: anti-inflammatory (suppressive) T regulatory (Treg) cells and proinflammatory Th17 cells [2,3].

CD4^+^ Treg cells can be divided into natural regulatory T cells (nTreg cells) and inducible or adaptive regulatory T cells (iTreg cells) [4]. nTreg got their name because they are always present in the body and constantly perform their function during normal surveillance of self-antigens [4]. They arise in thymus and leave it fully active, ready to inhibit immunological responses. They constitute the population of long-living cells, resident in peripheral organs and one of their main activities is protection against autoimmunological disorders [4]. The first described marker of these cells was CD25 with a more recent marker of nTreg cells being the constitutively expressed transcription factor Foxp3 [4]. Therefore CD4^+^Foxp3^+^ or CD4^+^CD25^+^Foxp3^+^ cells are often regarded as nTreg cells, in spite of the fact that newer markers have also been proposed [5]. However it should be noted that we still lack precise and specific markers of nTreg cells [6].

iTreg, in contrast to nTreg, obtain their regulatory ability in peripheral tissues. Naïve or mature CD4^+^ cells are activated by dendritic cells in the secondary lymphoid organs [7]. The main function of iTreg is therefore to counteract excessive or the pathological inflammatory response [7,8]. There are no good surface markers of iTreg and according to some authors they lack CD25 expression (CD4^+^CD25^-^ cells) [8] whereas others have shown that CD4^+^CD25^+^ iTreg can be generated from naïve CD4^+^ cells after antigen stimulation in the presence of IL-2 and TGF-β [9]. Furthermore it was shown that TGF-β-induced iTreg cells may express Foxp3 [10] and that Foxp3^+^ Treg cells are a rich source of TGF-β [11-13]. These latter cells are also known as Th3 cells. Phenotypic characterization of Th3 cells is very difficult as not all TGF-β-secreting T cells are Th3 cells and not all Th3 cells express Foxp3 [11-13].

Tr1 cells are another subset of iTreg cells, which is difficult to study in great details as we still lack precise markers, which could help us to reliably distinguish IL-10-secreting Treg cells from other T cells [14]. Tr1 cells produce IL-10, TGF-β and little or no IL-2, IL-4 and can be sometimes defined as: IL-10^+^IL-5^+^IL-4^-^IL-2^+/-^IFN-γ^+^. Many studies have demonstrated that IL-10-secreting T cells did not express Foxp3 [15-17] but there are also reports of subsets of Foxp3-expressing Treg cells with the ability to secrete IL-10 [18,19]. Phenotypic characterization of Tr1 cells could be therefore defined as CD4^+^IL-10^+^IL-4^-^.

The percentage of CD4^+^CD25^high^ cells was decreased in bronchoalveolar lavage (BAL) fluid of pediatric asthma patients [20]. Also Foxp3 protein expression was decreased within blood CD4^+^CD25^high^ cells [21]. However, Smith et al. have recently shown an increased percentage of CD4^+^Foxp3^+^ and CD4^+^CD25^+^CD127^-^ cells in BAL fluid of moderate to severe asthma [22]. In another study the percentage of CD4^+^Foxp3^+^ cells in BAL increased after allergen provocative test in asthmatic patients [23].

Th17 cells are a new population of proinflammatory CD4+ T cells which are important during activation of the inflammatory response against bacterial infection for example [24]. Th17 cells have an opposite function to Treg cells (especially iTreg cells) in relation to Th1 and Th2 cells. Interestingly, TGF-β in the presence of IL-6 induced the development of Th17 cells from naïve T cells whereas TGF-β alone induced the development of CD4^+^CD25^+^Foxp3^+^ Treg cells [25,26]. Mouse Th17 cells produce large amounts of IL-17, but no IL-4, IFN-γ or Foxp3 and contribute to airway hyper-responsiveness [25,27]. Several studies showed a possible involvement of Th17 cells in the pathogenesis of asthma. The number of IL-17 positive cells in sputum and BAL as well as the expression of mRNA for IL-17 in sputum cells is increased in asthma [28,29]. The levels of IL-17 in sputum correlated with hyperresponsiveness to methacholine in asthmatic subjects [30]. Increased levels of IL-17 in sputum were also seen in patients with allergic rhinitis after a nasal allergen challenge [31]. Interestingly, two recent reports showed the opposite results [32,33]. In the first study an increased expression of IL-17 in the bronchial epithelium was observed in severe asthma but not in mild or moderate asthma [32] whereas increased expression of IL-17 was reported in mild-moderate, but not severe asthma, in the second study [33].

In this study we isolated CD4^+^ cells from BAL fluid of asthmatics and age-matched control subjects and after short *in vitro* stimulation we measured simultaneously the expression of up to six parameters in these cells:

set 1: TGF-β, FoxP3, CD25, IFN-γ, IL-4, TNF-α;

set 2: IL-10, FoxP3, CD25, IFN-γ, IL-4, MIP-1β;

set 3: IL-17A, IL-8, IFN-γ, IL-4, MIP-1β.

The aim of our study was to analyze the incidence of different subsets of CD4^+^ T cells in BAL fluid of asthmatics and healthy subjects, in particular CD4^+^CD25^+^Foxp3^+^ cells, CD4^+^Foxp3^+^ cells, CD4^+^IL-10^+^IL-4^-^ cells, CD4^+^TGF-β^+^ cells, CD4^+^TGF-β^+^Foxp3^+^ cells and CD4^+^IL-17^+^ cells together with the co-expression of various cytokines: IFN-γ - a marker of Th1 cells, IL-4 – a marker of Th2 cells, IL-8 (CXCL8) – a chemokine for neutrophils, MIP-1β (CCL4) – a chemokine for monocytes and macrophages and TNF-α - a proinflammatory cytokine.

## Materials and methods

### Subjects

The study protocol was approved by the ethics committee of the Silesian Medical University. All subjects gave written consent before recruitment to the study. Twenty five stable asthmatic patients with a median age of 50 years (range 24–69 years) were recruited to the study. The inclusion criteria were as follow: the presence of consistent history of asthma together with objective evidence of asthma such as: reversible of airflow obstruction (improvement in FEV_1_ greater than 12% or 200 ml 15 minutes after administration of 200 μg of inhaled salbutamol) or airway hyperresponsiveness to methacholine (PC_20_ < 8 mg/ml). All patients were treated with inhaled steroids (100–1000 μg/day of fluticasone or equivalent).

The control group consisted of 12 non atopic age-matched healthy subjects with a median age of 42 years (range 26–62 years) with no history of any respiratory disease and no evidence of variable airflow obstruction or airway hyperresponsiveness.

The exclusion criteria for both studied groups were respiratory infection or use of oral corticosteroids within 6 weeks before entering the study. Current and ex-smokers of >5 pack-years were also excluded from the study. Subject characteristics are shown in Table 1.

**Table 1:** Characteristics of study subjects

**Group**	**Patients with asthma**	**Control subjects**
N	25	12
Age (y)	50 (24–69)	42 (26–62)
Sex (M/F)	8/17	10/2
FEV_1_ (% predicted)	86.6 (45.6–122.1)‡	103.7 (95.1–123.5)
FEV_1_/FVC (%)	72.4 (48.9–87.4)‡	85.6 (75.0–94.7)

Data are expressed as median (range).

M, Male; F, female; FEV_1_, forced expiratory volume in 1 sec; FVC, forced vital capacity.

‡*P* < .001 compared with the control group.

### Bronchoscopy and BAL

The tip of the bronchoscope was wedged into the orifice of a subsegmental bronchus of the middle lobe. Bronchoalveolar lavage was done by sequential installation and aspiration of 8 separate aliquots of 25 mL of warm 0.9% normal saline.

### Isolation of CD4+ T cells from BAL fluid

CD4+ T cells were isolated from BAL fluid as previously described [34]. Briefly, BAL cells were spun, washed and resuspended in culture medium (RPMI-1640, 10% [vol/vol] FCS, 2 mM l-glutamine, 100 IU/mL penicillin, and 100 mg/mL streptomycin). Macrophages were depleted from cells by means of adhesion to plastic plates. Resulting nonadherent cells consisted mainly of lymphocytes, but variable amounts of dead cells, granulocytes, and epithelial cells were also present. Dead cells were eliminated with the Dead Cell Removal Kit (Miltenyi Biotec, Bergisch Gladbach, Germany). Subsequently, CD8+ T cells were removed by using MACS CD8+ MicroBeads (Miltenyi Biotec). Finally CD4+ T cells were isolated by positive selection using MACS CD4+ MicroBeads. The purity of isolated CD4+ T cells were consistently >95% as assessed by FACS.

### Flow cytometry

CD4+ cells were washed and stimulated for 6 hours with phorbol 12-myristate 13-acetate (PMA; 10 ng/mL) and ionomycin (400 ng/mL) in the presence of brefeldin A (5 mg/mL). Cells were then washed twice in wash buffer, fixed in cold 4% formaldehyde, washed in PBS, and frozen at -70°C for later use as described previously [34]. Subsequently, cells were thawed, washed, permeabilized, washed again and stained with a cocktail of antibodies (described in details in Table 2) or respective mouse isotype control antibodies for 30 minutes at room temperature in the dark (all antibodies were from BD Pharmingen, Oxford, UK with exception for PE-labeled anti-TGF-β purchased at IQ Products, Groningen, The Netherlands). After being washed twice, the cells were resuspended in 1% formaldehyde in PBS and analyzed within 24 hours by means of six-colour flow cytometry (FACSAria flow cytometer, BD Biosciences). Representative plots of flow cytometric analysis were shown in Additional file 1.

**Table 2:** The sets of antibodies used in the study

**Fluorochrome**	**Set Nr. 1**	**Set Nr. 2**	**Set Nr. 3**
FITC	Anti- IFN-γ	Anti- IFN-γ	Anti- IFN-γ
PE	Anti- TGF-β	Anti- FoxP3	Anti- IL-8
PerCP-Cy5.5	Anti- TNF-α	Anti- MIP-1β	Anti- MIP-1β
PE-Cy7	Anti- IL-4	Anti- IL-4	Anti- IL-4
APC or Alexa Fluor 647	Anti- FoxP3	Anti- IL-10	Anti- IL-17A
APC-Cy7	Anti- CD25	Anti- CD25	

FITC - Fluorescein isothiocyanate; PE - R-phycoerythrin; PerCP-Cy5.5 - a tandem fluorochrome composed of peridinin chlorophyll protein (PerCP) coupled to the cyanine dye Cy5.5; PE-Cy7 - a tandem fluorochrome composed of R-phycoerythrin (PE) coupled to the cyanine dye Cy7; APC - Allophycocyanin; APC-Cy7 - a tandem fluorochrome composed of Allophycocyanin (APC) coupled to the cyanine dye Cy7.

### Statistical analysis

Statistical analysis was performed using software packages (GraphPad Prism 5.0; Statistica 9.0). Data are presented as median (95% confidence intervals) except where stated. A Gaussian distribution was tested with D’Agostino and Pearson omnibus normality test. Between-group comparisons were assessed using a Mann–Whitney *U* test for non-parametric data. Regression analysis was performed using the Spearman rank correlation test. *P* values of less than 0.05 were accepted as significant.

## Results

### BAL cell characterization

The percentage of eosinophils in BAL fluid of asthmatics was significantly higher compared to control subjects (p < 0.01). Full characterisation of BAL cells is provided in Table 3.

**Table 3:** Characteristics of BAL cells from the study population

**Group**	**Patients with asthma**	**Control subjects**
Recovery (ml)	122 (90–147)	123.5 (95–140)
Total cells number (×10^6^)	3.9 (0.7–32.8)	5.0 (1.4–27.4)
Total cell number per volume of BAL recovered (×10^4^/ml)	3.0 (0.7–25.0)	4.1 (1.0–23.2)
Macrophages (%)	89.0 (41.5–97.8)	91.7 (82.7–98.7)
Neutrophils (%)	0.5 (0–3.9)	0.3 (0–1.7)
Eosinophils (%)	0.3 (0–10.0)†	0 (0–0.7)
Lymphocytes (%)	10.2 (1.8–57.3)	7.9 (1.3–16.4)

Data are expressed as median (range).

†*P* < .01 compared with the control group.

### Percentage of BAL CD4^+^ cells expressing and co-expressing Foxp3

**Figure 1:**
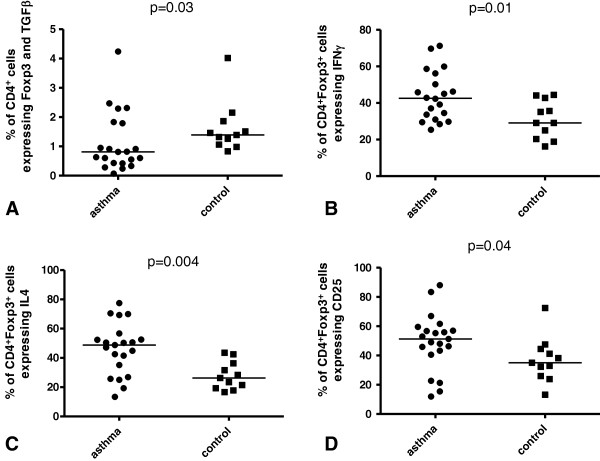
**Percentage of BAL CD4**^**+ **^**cells expressing and co-expressing Foxp3.** The percentage of **(A)** BAL CD4^+^ cells co-expressing Foxp3 and TGF-β (CD4^+^Foxp3^+^TGF-β^+^ cells), **(B)** CD4^+^Foxp3^+^ BAL cells expressing IFN-γ, **(C)** CD4^+^Foxp3^+^ BAL cells expressing IL-4 and **(D)** CD4^+^Foxp3^+^ BAL cells expressing CD25.

The percentage of CD4^+^ cells co-expressing Foxp3 and TGF-β (CD4^+^Foxp3^+^TGF-β^+^ cells) was significantly lower in asthmatics [0.8% (0.1–4.2%)] compared with that seen in healthy subjects [1.4% (0.8–4.0%), Figure 1A; *P* = 0.03]. In contract, there was no significant differences between asthma and control groups in the percentage of CD4^+^ cells expressing Foxp3 (CD4^+^Foxp3^+^ cells) [4.4% (0.8–12.5%) vs 6.8% (2.1–12.6%)], CD4^+^Foxp3^+^IFN-γ^+^ cells [2.1% (0.3–5.6%) vs 1.8% (0.6–3.4%)], CD4^+^Foxp3^+^TNF-α^+^ cells [1.6% (0.4–4.3%) vs 1.8% (0.8–5.4%)], CD4^+^Foxp3^+^IL-4^+^ cells [1.9% (0.2–8.7%) vs 1.6% (0.4–4.2%)], CD4^+^Foxp3^+^IL-10^+^ cells [2.0% (0.1–7.5%) vs 1.1% (0.3–5.7%)] or CD4^+^Foxp3^+^CD25^+^ cells [2.3% (0.1–10.7%) vs 2.6% (0.7–4.3%)].

A significantly higher percentage of CD4^+^Foxp3^+^ cells from asthma patients compared with healthy subjects expressed IFN-γ [42.6% (25.4–71.2%) vs 29.1% (16.2–44.4%); Figure 1B; *P* = 0.01], IL-4 [48.8% (13.4–77.4%) vs 26.3% (16.7–43.6%); Figure 1C; *P* = 0.004] and CD25 [51.3% (11.9–88.1%) vs 35.1% (13.2–72.5%); Figure 1D; *P* = 0.04). In contrast, no significant difference between the studied groups were observed for the percentage of CD4^+^Foxp3^+^ cells expressing TGF-β [18.2% (4.9–49.3%) vs 25.7% (11.4–46.5%)], IL-10 [35.9% (3.6–61.5%) vs 19.6% (3.3–52.5%); *P* = 0.07] or TNF-α [33.7% (10.9–80.9%) vs 33.0% (11.0–66.7%)].

### Percentage of BAL CD4^+^ cells expressing and co-expressing TGF-β

The percentage of CD4^+^TGF-β^+^ cells [3.0% (0.5–11.6%) vs 4.1% (0.7–12.9%)], CD4^+^TGF-β^+^CD25^+^ cells [0.4% (0–1.8%) vs 0.5% (0.1–1.7%)], CD4^+^TGF-β^+^IL-4^+^ cells [0.5% (0.1–2.7%) vs 0.4% (0–2.8%)], CD4^+^TGF-β^+^IFN-γ^+^ cells [1.6% (0.3–5.9%) vs 1.4% (0.2–5.1%)] or CD4^+^TGF-β^+^TNF-α^+^ cells [2.2% (0.3–4.9%) vs 1.2% (0.3–4.4%)] did not significantly differ between asthma and control groups.

**Figure 2:**
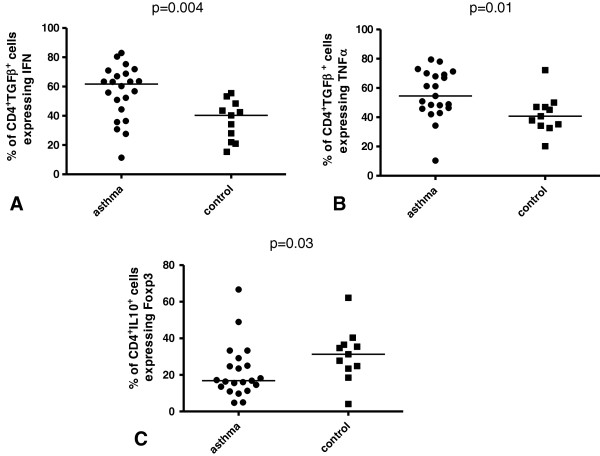
**Percentage of BAL CD4**^**+ **^**cells expressing and co-expressing TGF-β or IL-10.** The percentage of BAL CD4^+^TGF-β^+^ cells expressing **(A)** IFN-γ and **(B)** expressing TNF-α. **(C)** The percentage of BAL CD4^+^IL-10^+^ cells expressing Foxp3.

CD4^+^TGF-β^+^ cells from asthmatics compared with healthy subjects expressed significantly more IFN-γ [61.7% (11.4–82.9%) vs 40.3% (15.4–55.5%); Figure 2A; *P* = 0.004] and TNF-α [54.6% (10.4–79.5%) vs 40.7% (20.2–72.2%); Figure 2B; *P* = 0.01]. The expression of Foxp3 [28.1% (4.9–61.0%) vs 36.4% (13.7–52.9%)], CD25 [14.3% (0–28.8%) vs 14.9% (7.9–28.9%)] or IL-4 [16.9% (3.5–37.3%) vs 10.7% (0–34.0%)] in CD4^+^TGF-β^+^ cells did not differ significantly between asthma and control groups.

### Percentage of BAL CD4^+^ cells expressing and co-expressing IL-10

There were no significant difference between asthmatics and healthy subjects concerning the percentage of CD4^+^IL-10^+^IL-4^-^ cells [1.6% (0.2–4.0%) vs 1.3% (0.4–9.5%)], CD4^+^IL-10^+^ cells [3.6% (0–18.6%) vs 3.8% (0.9–14.3%)], CD4^+^IL-10^+^CD25^+^ cells [1.6% (0.1–15.9%) vs 1.6% (0.7–9.0%)], CD4^+^IL-10^+^IL-4^+^ cells [1.8% (0.1–7.2%) vs 1.1% (0.3–1.7%)], CD4^+^IL-10^+^ IFN-γ^+^ cells [1.6% (0–7.8%) vs 1.0% (0.3–3.6%)] or CD4^+^IL-10^+^MIP-1β^+^ cells [1.8% (0.1–7.2%) vs 1.1% (0.3–1.7%)]. In contrast, the percentage of CD4^+^IL-10^+^ cells expressing Foxp3 was significantly decreased in asthmatics compared with healthy subjects [16.9% (4.8–66.7%) vs 31.3% (4.1–62.2%); Figure 2C; *P* = 0.03]. No differences between the two groups were observed for the expression of CD25 [54.0% (13.1–95.0%) vs 62.9% (21.5–77.5%)], IL-4 [56.7% (11.5–92.2%) vs 52.3% (22.8–71.7%)], IFN-γ [37.4% (2.4–72.4%) vs 29.1% (7.2–71.2%)] or MIP-1β [39.1% (12.1–76.5%) vs 24.0% (11.9–65.2%)] in CD4^+^IL-10^+^ cells.

### Percentage of BAL CD4^+^ cells expressing and co-expressing IL-17A

**Figure 3:**
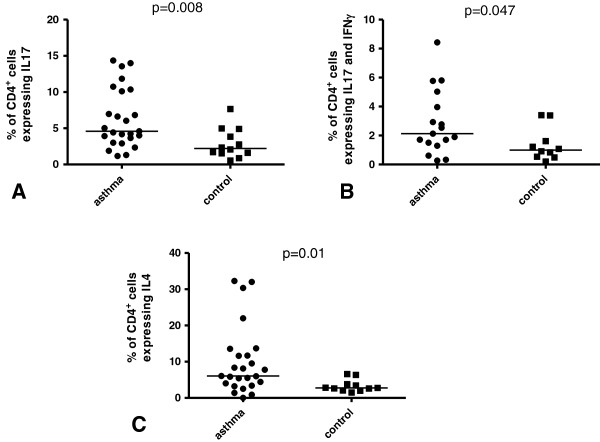
**Percentage of BAL CD4**^**+ **^**cells expressing and co-expressing IL-17A or IL-4.** The percentage of BAL **(A)** CD4^+^ cells expressing IL-17 (CD4^+^IL-17^+^ cells), **(B)** CD4^+^ cells co-expressing IL-17 and IFN-γ (CD4^+^IL-17^+^ IFN-γ^+^ cells) and **(C)** CD4^+^ cells expressing IL-4 (CD4^+^IL-4^+^ cells).

The percentage of CD4^+^IL-17^+^ cells [4.6% (1.2–14.4%) vs 2.2% (0.5–7.7%); Figure 3A; *P* = 0.008] and CD4^+^IL-17^+^ IFN-γ^+^ cells [2.1% (0.3–8.4%) vs 1.0% (0.2–3.4%); Figure 3B; *P* = 0.047] were significantly increased in asthmatics compared with that seen in healthy subjects. No significant differences between the two groups were observed for the percentage of CD4^+^IL-17^+^IL-8^+^ cells [1.4% (0.2–7.5%) vs 0.8% (0.2–2.2%); *P* = 0.09], CD4^+^IL-17^+^MIP-1β^+^ cells [1.6% (0.2–7.5%) vs 0.5% (0.1–4.1%); *P* = 0.07] or CD4^+^IL-17^+^IL-4^+^ cells [0.5% (0–2.5%) vs 0.3% (0.1–0.6%)]. Similarly, the percentage of CD4^+^IL-17^+^ cells expressing IFN-γ [50.0% (22.7–65.6%) vs 44.4% (33.6–68.3%)], IL-8 [36.5% (15.8–72.3%) vs 31.9% (12.7–72.1%)], IL-4 [10.1% (0–33.3%) vs 9.3% (1.4–37.5%)] or MIP-1β [33.5% (5.7–75.3%) vs 32.6% (14.1–68.6%)] did not differ between the two groups.

### Percentage of BAL CD4^+^ cells expressing other cytokines and markers

Significantly increased percentage of CD4^+^ cells expressing IL-4 (CD4^+^IL-4^+^ cells) was seen in asthma patients [6.1% (0–32.3%)] compared with healthy subjects [2.8% (1.5–6.6%), Figure 3C; *P* = 0.01]. No further statistically significant differences concerning the percentage of CD4^+^ cells expressing IFN-γ [19.2% (7.3–49.8%) vs 17.0% (4.8–29.8%)], IL-8 [9.7% (1.0–23.4%) vs 9.4% (3.2–17.4%)], TNF-α [29.8% (2.5–75.2%) vs 15.4% (3.3–58.3%)], MIP-1β [11.8% (1.1–58.5%) vs 9.7% (2.6–33.4%)] or CD25 [4.3% (0.4–17.5%) vs 4.9% (1.7–16.3%)] were observed between asthma and control groups.

### Correlation between different subtypes of CD4^+^ cells and spirometry results or the percentage of BAL cells

**Figure 4:**
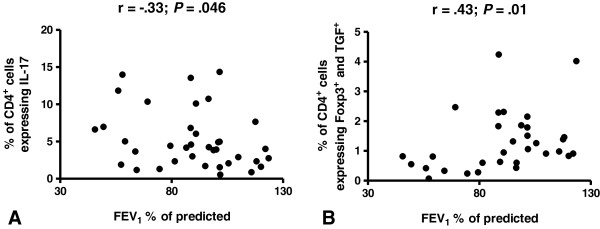
**Correlation between different subtypes of CD4**^**+ **^**cells and spirometry results or the percentage of BAL cells. A**. CD4^+^IL-17^+^ cells vs. FEV_1_% predicted. **B**. CD4^+^Foxp3^+^TGF-β^+^ cells vs. FEV_1_% predicted.

The percentage of CD4^+^IL-17^+^ cells correlated negatively with FEV_1_% predicted (r = -0.33; *P* < 0.05; Figure 4A), whereas the percentage of CD4^+^Foxp3^+^ cells showed a positive correlation with FEV_1_/FVC (r = 0.39; *P* < 0.05). Similarly, CD4^+^Foxp3^+^TGF-β^+^ cells correlated positively with FEV_1_% predicted (r = 0.43; *P* < 0.05; Figure 4B) and FEV_1_/FVC (r = 0.57; *P* < 0.05) but negatively with the percentage of lymphocytes in the BAL fluid (r = -0.50; *P* < 0.05), the number of CD4^+^ cells (r = -0.53; *P* < 0.05) and the number of CD8^+^ cells (r = -0.62; *P* < 0.05) in BAL fluid. We also observed that the percentage of CD4^+^TGF-β^+^CD25^+^ cells correlated negatively with the number of CD4^+^ cells (r = -0.39; *P* < 0.05) and the number of CD8^+^ cells (r = -0.44; *P* < .05) in BAL fluid.

## Discussion

In this study we analyzed the incidence of different subsets of CD4^+^ T cells together with the co-expression of various cytokines in BAL fluid of asthma patients and healthy subjects. We found that in BAL fluid of patients with chronic asthma have a decreased population of CD4^+^Foxp3^+^TGF-β^+^ cells and at the same time an increased population of CD4^+^IL-17^+^ cells, CD4^+^IL-17^+^ IFN-γ^+^ cells and CD4^+^IL-4^+^ cells. We also found that decreasing FEV_1_ correlated with the decreasing percentage of CD4^+^Foxp3^+^TGF-β^+^ cells and simultaneously with the increasing percentage numbers of CD4^+^IL-17^+^ cells. Additionally, CD4^+^Foxp3^+^ cells derived from the BAL fluid of asthmatics were characterized by an increased expression of IFN-γ, IL-4 and CD25, whereas CD4^+^IL-10^+^ cells had a decreased expression of Foxp3.

Our study is the first to show the decreased population of CD4^+^Foxp3^+^TGF-β^+^ cells in BAL fluid of asthma patients. We did not observe a significant difference between asthma and controls with respect to the incidence of other Foxp3 or IL-10 positive cells: CD4^+^Foxp3^+^, CD4^+^Foxp3^+^CD25^+^, CD4^+^IL-10^+^IL-4^-^ or CD4^+^Foxp3^+^IL-10^+^. Therefore the results of our study do not confirm either the recent report of Smith et al., who showed increased percentage of CD4^+^Foxp3^+^ and CD4^+^CD25^+^CD127^-^ cells in BAL fluid of moderate to severe asthma [22] or the results of Hartl et al. who found a decreased percentage of CD4 + CD25^high^ cells in BAL fluid of pediatric asthma patients [20]. Hartl and colleagues also found that the suppressive capacity of CD4 + CD25^high^ cells in BAL fluid was depended on TGF-β, so they speculated that these cells may represent iTreg cells. The results of our study are therefore much closer to those of Hartl et al. as we showed a decreased percentage of CD4^+^Foxp3^+^TGF-β^+^ cells in BAL fluid of asthma patients. Similarly, we speculate that this subset of T cells may represent Th3 iTreg cells as these cells express TGF-β. Additionally, the percentage of CD4^+^Foxp3^+^CD25^+^ did not differ between the two groups studied.

Asthma is an inflammatory disease of a peripheral organ, the lung, so the involvement of locally-induced iTreg cells instead of nTreg cells in the pathogenesis of this disease seems to be more probable. The decreased population of Th3 iTreg cells may, therefore, contribute to the presence of airway inflammation and to asthma progression since there is a correlation between the percentage of CD4^+^Foxp3^+^TGF-β^+^ cells and FEV_1_.

Although several studies have previously shown the importance of IL-17 and IL-17 producing cells in asthma pathogenesis our study is, to our knowledge, the first to show using flow cytometry and intracellular cytokine staining that the percentage of CD4^+^IL-17^+^ cells in BAL fluid of asthma patients is increased. Th17 cells are known inducers of neutrophilic inflammation as they stimulate other cells (e.g. epithelial cells) to produce IL-8/CXCL8, which is one of the most potent neutrophil chemoatractants [2]. In this study we showed that CD4^+^IL-17^+^ cells in humans are able to produce IL-8/CXCL8, but we observed no differences in the percentage of CD4^+^IL-17^+^IL-8^+^ cells between asthmatic and control groups. Th17 cells may also cooperate with Th2 cells to induce eosinophilic airway inflammation in mice [35]. In our study we confirmed an increased population of Th2 cells, which may indicate that Th17 and Th2 may work together to play an important role in the pathogenesis of human asthma.

The role of CD4^+^IL-17^+^ cells in pathogenesis of asthma is further supported by the presence of a negative correlation with FEV_1_. Interestingly, approximately half of the CD4^+^IL-17^+^ cells in our study expressed INF-γ^+^ and the percentage of these CD4^+^IL-17^+^INF-γ^+^ cells was significantly increased in asthma (*P* = 0.047). Th17 cells show great degree of plasticity and during the inflammatory response Th17/Th1 cells can be readily detected [36] indicating a close relationship between Th1 and Th17 cell differentiation programs [37,38]. Our results suggest therefore that part of increased activity of Th17 cells in asthma could be attributed to Th17/Th1 cells.

Although we observed no difference in the percentage of CD4^+^IL-10^+^, CD4^+^IL-10^+^IL-4^-^ or CD4^+^IL-10^+^Foxp3^+^ cells between the asthma and control groups, we found that CD4^+^IL-10^+^ cells of asthmatics expressed less Foxp3. Many studies have shown that IL-10-secreting Treg cells (Tr1) generally do not express Foxp3, but IL-10-secreting Foxp3^+^CD4^+^ cells have also been reported by others. Our findings suggest therefore that Foxp3^-^ IL-10-secreting CD4^+^ cells predominate in asthmatic patients.

Increased expression of IFN-γ and IL-4 by CD4^+^Foxp3^+^ cells will suppress the immune response in asthmatic patients. nTreg are poor producers of proinflammatory cytokines such as IL-4, IFN-γ or IL-17 [17]. However, recent studies indicate that a subset of CD4^+^Foxp3^+^ cells in humans are not regulatory but effector CD4^+^ cells [39-41]. It may be possible, therefore, that CD4^+^Foxp3^+^ cells expressing IFN-γ or IL-4 represent activated effector CD4^+^ cells. Alternatively, since iTreg cells show much higher level of plasticity than nTreg cells [37,38] and are able to transform into CD4^+^Foxp3^+^IL-17^+^ cells in the presence of an inflammatory cytokine milieu [42]. A similar conversion of Foxp3^+^ Treg cells into effector cells secreting IL-4 or IFN-γ has been observed and these cells were able to express low levels of Foxp3 [43,44]. It may therefore possible that a subset of CD4^+^Foxp3^+^ cells expressing IL-4 or IFN-γ cells are iTreg cells. Additionally, the increased population of CD4^+^Foxp3^+^IFN-γ^+^ and CD4^+^Foxp3^+^IL-4^+^ cells in asthmatics suggest that CD4^+^Foxp3^+^ cells in those patients are very plastic and depending on different circumstances may show different phenotypes.

In conclusion, our results suggest that in the airways of chronic asthma patients there is an imbalance between the increased population of CD4^+^IL-17^+^ cells, CD4^+^IL-17^+^IFN-γ^+^ cells and CD4^+^IL-4^+^ cells and the decreased population of CD4^+^Foxp3^+^TGF-β^+^ cells. This imbalance may be important for development of airway obstruction in asthma as we found that decreased FEV_1_ correlated with the decreasing percentage of CD4^+^Foxp3^+^TGF-β^+^ cells and simultaneously with the increasing percentage numbers of CD4^+^IL-17^+^ cells.

## Abbreviations

BAL: Bronchoalveolar lavage; CCL4: CC chemokine ligand 4; CD: Cluster of differentiation; CXCL8: CXC chemokine ligand 8; FCS: Fetal calf serum; FEV_1_: Forced expiratory volume in 1 second; FoxP3: Forkhead box P3; FVC: Forced vital capacity; IFN-γ: Interferon-γ; IL: Interleukin; iTreg: Inducible T regulatory; MIP-1β: Macrophage inflammatory protein-1β; nTreg: Natural T regulatory; PC_20_: Provocative concentration causing a 20% fall in FEV_1_; PMA: Phorbol 12-myristate 13-acetate; TGF-β: Tranforming growth factor-β; Th: T helper; TNF-α: tumor necrosis factor-α; Tr1: T regulatory type 1; Treg: T regulatory.

## Competing interests

All authors declare that they have no competing interests.

## Authors’ contributions

AB, WP, MK, PJB, IMA defined the research theme; AB, WP, IMA designed the study, AB, GC, RW, IMA designed methods and experiments, AB, WP, collected subjects’ samples, AB, RW, carried out the laboratory experiments, AB, WP, RW, MK, IMA participated in analysis of data, AB, GC, MK, IMA performed the statistical analysis, AB, WP, RW, PJB, IMA interpreted the results, and AB, WP, GC, RW, MK, PJB, IMA wrote the paper. All authors read and approved the manuscript.

## Supplementary Material

Additional file 1: Figure S1 Representative plots of flow cytometric analysis. (A) BAL CD4+ cells expressing or co-expressing TGF-β or Foxp3 from asthma patient. (B) BAL CD4+ cells expressing or co-expressing TGF-β or Foxp3 from control subject. (C) BAL CD4+ cells expressing or co-expressing IFN-γ or IL-17 from asthma patient. (D) BAL CD4+ cells expressing or co-expressing IFN-γ or IL-17 from control subject. Cells were stimulated with PMA/ionomycin for 6 hrs in the presence of Brefeldin A. The plots are representative of those from 25 asthmatic and 12 healthy control subjects. Click here for file
