# Supplementary figures and images for: Decreased percentage of CD4+Foxp3+TGF-β+ and increased percentage of CD4+IL-17+ cells in bronchoalveolar lavage of asthmatics
Source: J Inflamm (Lond). 2014 Aug 9;11:22. doi: 10.1186/1476-9255-11-22 (PMC4133956; doi:10.1186/1476-9255-11-22)

**Fig S1A**

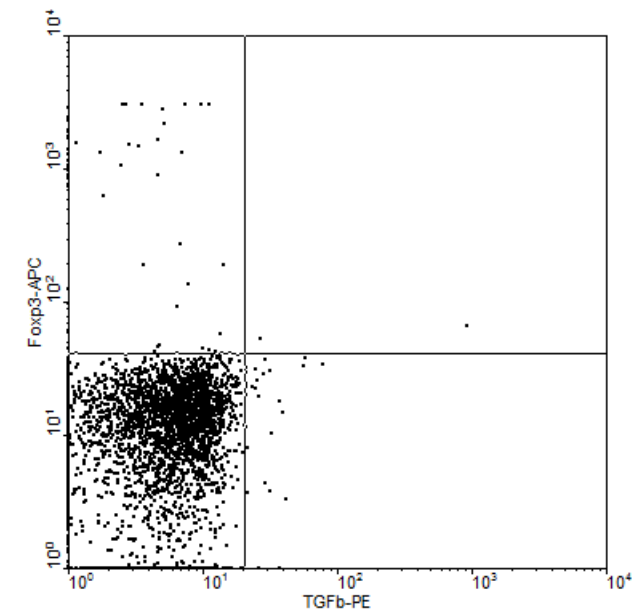

**Fig S1B**

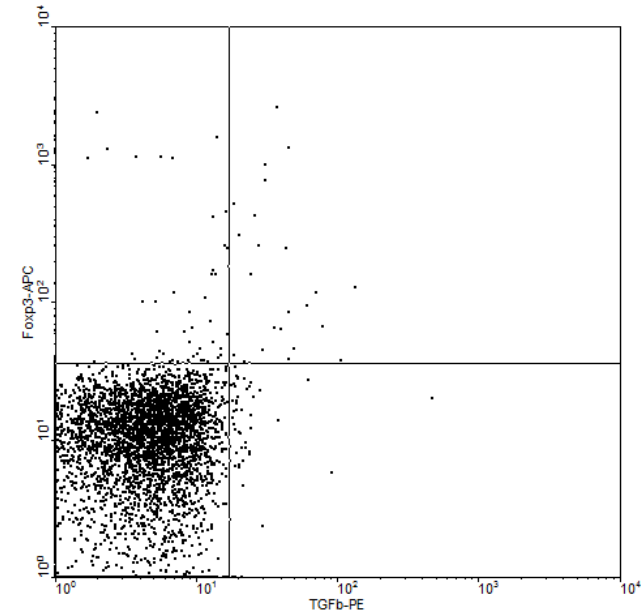

**Fig S1C**

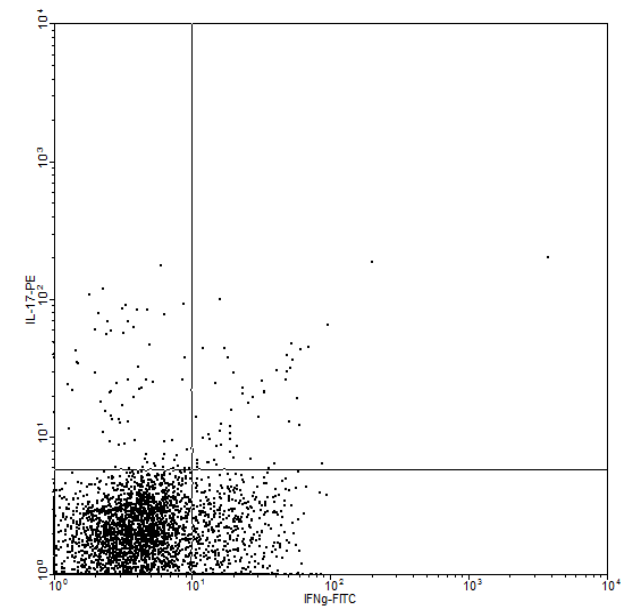

**Fig S1D**

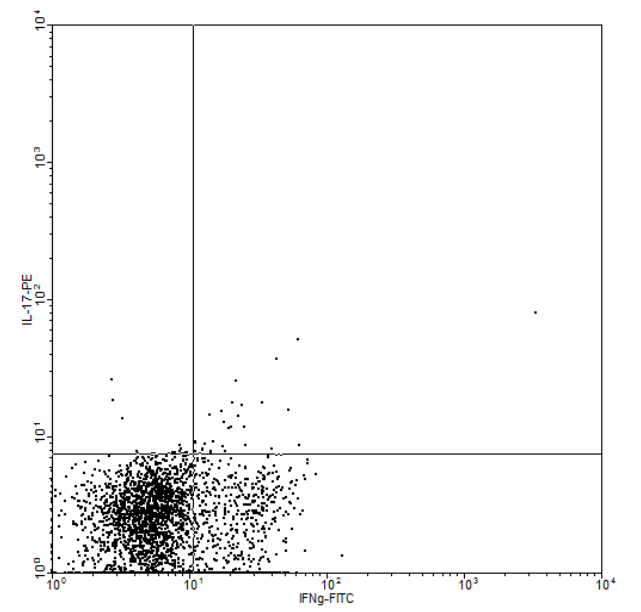

Supplement: Additional file 1: Figure S1 — Representative plots of flow cytometric analysis. (A) BAL CD4+ cells expressing or co-expressing TGF-β or Foxp3 from asthma patient. (B) BAL CD4+ cells expressing or co-expressing TGF-β or Foxp3 from control subject. (C) BAL CD4+ cells expressing or co-expressing IFN-γ or IL-17 from asthma patient. (D) BAL CD4+ cells expressing or co-expressing IFN-γ or IL-17 from control subject. Cells were stimulated with PMA/ionomycin for 6 hrs in the presence of Brefeldin A. The plots are representative of those from 25 asthmatic and 12 healthy control subjects. [file 1476-9255-11-22-S1.pdf]
